# *S*-acylation in apoptotic and non-apoptotic cell death: a central regulator of membrane dynamics and protein function

**DOI:** 10.1042/BST20253012

**Published:** 2025-04-29

**Authors:** Rojae Manhertz-Patterson, G. Ekin Atilla-Gokcumen

**Affiliations:** Department of Chemistry, University at Buffalo, The State University of New York, Buffalo, New York 14260, U.S.A.

**Keywords:** apoptosis, cell death, necroptosis, palmitoylation, pyroptosis, *S*-acylation

## Abstract

Protein lipidation is a collection of important post-translational modifications that modulate protein localization and stability. Protein lipidation affects protein function by facilitating interactions with cellular membranes, changing the local environment of protein interactions. Among these modifications, S-acylation has emerged as a key regulator of various cellular processes, including different forms of cell death. In this mini-review, we highlight the role of S-acylation in apoptosis and its emerging contributions to necroptosis and pyroptosis. While traditionally associated with the incorporation of palmitic acid (palmitoylation), recent findings indicate that other fatty acids can also participate in S-acylation, expanding its functional repertoire. In apoptosis, S-acylation influences the localization and function of key regulators such as Bcl-2-associated X protein and other proteins modulating their role in mitochondrial permeabilization and death receptor signaling. Similarly, in necroptosis, S-acylation of mixed lineage kinase domain-like protein (MLKL) with palmitic acid and very long-chain fatty acids enhances membrane binding and membrane permeabilization, contributing to cell death and inflammatory responses. Recent studies also highlight the role of S-acylation in pyroptosis, where S-acylated gasdermin D facilitates membrane localization and pore assembly upon inflammasome activation. Blocking palmitoylation has shown to suppress pyroptosis and cytokine release, reducing inflammatory activity and tissue damage in septic models. Collectively, these findings underscore S-acylation as a shared and important regulatory mechanism across cell death pathways affecting membrane association of key signaling proteins and membrane dynamics, and offer insights into the spatial and temporal control of protein function.

## Introduction

Protein lipidation is a crucial post-translational modification where lipid molecules are covalently attached to proteins, enhancing their hydrophobicity and targeting to cellular membranes. This modification not only affects membrane translocation but also affects structure, stability, and interactions with other proteins or lipids. By increasing membrane affinity, lipidation can regulate the spatial organization of proteins to various membrane-bound compartments within the cell, mediating localized cellular signaling. Additionally, the reversible nature of certain lipid modifications, such as palmitoylation, allows proteins to cycle between membrane-bound and cytosolic states, providing a dynamic mechanism for the temporal regulation of protein activity (reviewed in [1]).

**Figure 1:**
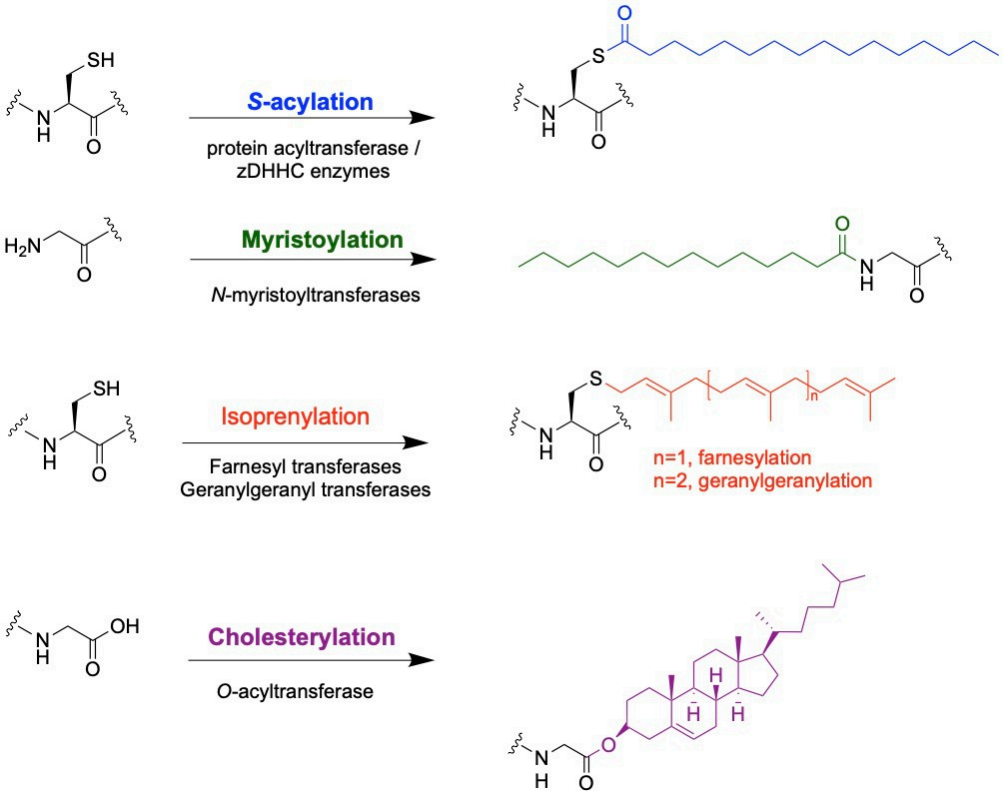
Major types of protein lipidation. *S*-acylation promoted by protein acyltransferases results in the covalent addition of fatty acids, shown in blue, to cysteine residues. Farnesyl and geranylgeranyl transferases mediated isoprenylation, involving the transfer of isoprene units (farnesyl diphosphate and geranylgeranyl diphosphatase, respectively), shown in red, to thiol groups present in cysteine residues near the C-terminal of proteins. *N*-myristoyltransferase catalyzed the transfer of myristic acid to the N-terminal glycine of proteins. C-terminal glycine residues can be modified with cholesterol via ester linkages, resulting in cholesterylation.

There are four major types of protein lipidation (reviewed in [2], Figure 1). *S*-acylation is one of the most prevalent lipidation mechanisms. It involves the covalent attachment of palmitic acid (C16:0 fatty acid) or other fatty acids to cysteine residues. This modification can be reversible and is dynamically regulated by other enzymes. Palmitoylation plays a crucial role in modulating the membrane affinity, subcellular localization, and trafficking of proteins, including G-proteins and G-protein-coupled receptors, thus influencing a variety of cellular processes and signaling pathways [1,2]. Myristoylation is another type of fatty acid modification and includes the irreversible modification of a myristic acid (C14:0 fatty acid) to the N-terminal glycine residue of target proteins. Membrane association of Src family kinases and G-protein subunits is controlled by myristoylation (Figure 1). In addition to palmitoylation and myristoylation, recent studies have shown other types of fatty acids can be used [3–5]. Prenylation involves the addition of farnesyl (C15) or geranylgeranyl (C20) isoprenoid groups to a cysteine proximal to the C-terminus of proteins. Prenylation is essential for anchoring proteins to cell membranes and is key to the function of signaling proteins like Ras and Rho GTPases (Figure 1). Lipids other than fatty acids and isoprenoids can also be incorporated into proteins, including glycosylphosphatidylinositol and cholesterol. Modification by cholesterol, known as cholesterylation, typically occurs at the C-terminal end of proteins and plays a crucial role in regulating their membrane association, trafficking, and signaling efficiency (Figure 1). It is most notably observed in proteins such as Hedgehog, a key signaling protein in embryonic development and tissue homeostasis [2].

Different types of lipidations impact many protein targets involved in diverse cellular processes (reviewed in [1,2,6]). In this review, we will focus on *S*-acylation and its critical impact on protein function in programmed cell death mechanisms. Specifically, we will highlight key signaling proteins whose activity is regulated by protein *S*-acylation, emphasizing the role of this modification in apoptosis, necroptosis, and pyroptosis. Additionally, we will explore emerging concepts in the field, such as the expanding repertoire of fatty acids that can be transferred by acyltransferase enzymes. Finally, we will provide insights into potential therapeutic strategies targeting protein *S*-acylation and discuss their implications for modulating cell death and other pathophysiological processes associated with this modification.

## *S*-Acylation is a common type of protein lipidation

*S*-Acylation is predominantly catalyzed by a family of enzymes known as protein acyltransferases (zDHHCs or PATs), which are characterized by a conserved DHHC (Asp-His-His-Cys) motif that forms a crucial part of the enzymes catalytic site and is essential to their ability to mediate the *S*-acylation reaction. This process can be reversible, which is facilitated by acyl-protein thioesterases (APTs) and palmitoyl-protein thioesterases. This dynamic regulation alters the protein’s membrane affinity and enables the modulation of subcellular localization and protein activity [1].

zDHHC proteins are localized in various cellular membranes, including the plasma membrane, Golgi apparatus, endoplasmic reticulum (ER), and endosomes. Their distribution allows them to modify a wide array of substrates in different cellular compartments, thus playing roles in various cellular processes like signal transduction and vesicle trafficking [7]. zDHHC5 and zDHHC20 are prominent examples of *S*-acyltransferases localized to the plasma membrane, while zDHHC3, zDHHC7, zDHHC9, zDHHC15, and zDHHC17 are primarily found in the Golgi apparatus. The Golgi localization enables these enzymes to play key roles in the palmitoylation of proteins destined for secretion or membrane trafficking, such as vesicular proteins and signaling molecules. zDHHC1, zDHHC6, zDHHC4, and zDHHC16 are mainly associated with the ER and participate in processes related to protein folding and quality control. Other zDHHCs, including zDHHC2, 17, and 13, can also be associated with endosomal and other vesicular compartments [8].

zDHHC enzymes exhibit substrate specificity influenced by factors such as cellular localization, interaction domains, and sequence motifs. While they share a conserved catalytic domain, their N- and C-terminal cytosolic domains, responsible for substrate recognition, exhibit significant diversity [2,5,7]. This substrate specificity can also be modulated by the cellular (co)localization of zDHHCs and their substrates, in addition to sequence motifs or structural domains within the target proteins. Studies using biorthogonal lipid analogs have demonstrated that individual zDHHCs can exhibit marked differences in fatty acid preferences and transfer efficiency [5]. The activity of zDHHCs can be regulated at multiple levels, including phosphorylation, ubiquitination, interactions with scaffolding proteins, and the lipid composition of the surrounding membrane, all of which can influence acyltransferase activity [9].

The use of small-molecule inhibitors that modulate acyltransferase or thioesterase activity has been instrumental in studying this modification [1,2], while most of these compounds exhibit limited specificity, highlighting opportunities for future research and tool development. As previously noted, the most common fatty acid observed in *S*-fatty acylation is C16:0 fatty acid. Recently, *S*-fatty acylation involving fatty acids of different chain lengths and degrees of unsaturation has also been reported. A global analysis of *S*-acylated proteins in platelets revealed that approximately 70% of the proteins were modified with C16:0 fatty acid, 22% with C18:0 fatty acid, and 4% with C18:1 fatty acid (oleic acid) [10]. Certain proteins, such as Src-family kinases [11] and mixed lineage kinase domain-like (MLKL) [3,4], can undergo heterogeneous *S*-fatty acylation, with the nature of the attached fatty acid influencing membrane dynamics and signaling outcomes. *In vitro* studies have shown that zDHHC2 utilizes a broad range of acyl-CoAs, with a preference for acyl chains of 14 carbons or longer, while zDHHC3 displays a strong preference for acyl chain lengths of 16 carbons or shorter [12]. Structural and biochemical assessments have provided some key observations regarding these preferences [5,13], though they are not covered in this review. The functional consequences of heterogeneous *S*-fatty acylation, as well as how zDHHCs utilize and are regulated by acyl-CoA pools, remain important areas for future investigation.

## The role of *S*-acylation in apoptotic and non-apoptotic programmed cell death

**Figure 2:**
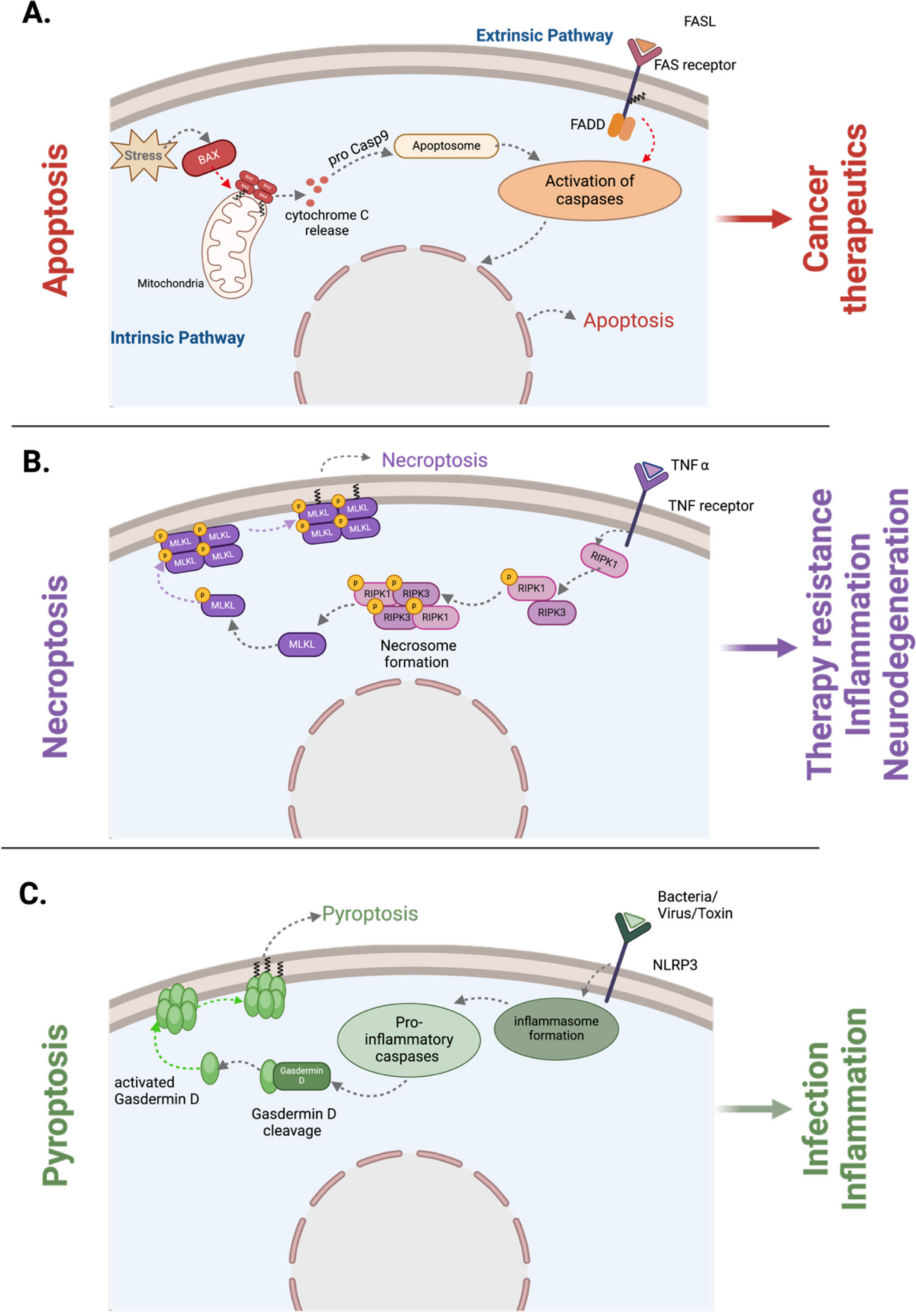
S-acylation in apoptosis, necroptosis and pyroptosis. (**A**) During the intrinsic apoptosis pathway, pro-apoptotic protein BAX is *S*-acylated at Cys126, facilitating its recruitment to the mitochondrial membrane. This recruitment enables BAX oligomerization, leading to mitochondrial membrane permeabilization. The permeabilized membrane allows cytochrome c release, caspase activation, and ultimately, apoptosis. Similarly, in the extrinsic pathway, the FAS receptor is *S*-acylated at Cys199, enhancing its membrane binding. This modification contributes to the activation of downstream caspases and triggers apoptosis. (**B**) In necroptosis, the formation of the phosphorylated RIPK1/RIPK3 necrosome initiates the recruitment and phosphorylation of MLKL. Phosphorylated MLKL undergoes selective S-acylation, which drives its translocation to the plasma membrane. At the membrane, this modification facilitates permeabilization and destabilization, leading to the loss of membrane integrity. This disruption contributes to the release of pro-inflammatory cytokines, a hallmark of necroptosis. (**C**) During pyroptosis, gasdermin D is cleaved by caspases, yielding its active form. The cleaved gasdermin D undergoes S-acylation, which enhances its ability to oligomerize and form pores in the plasma membrane. These pores exacerbate membrane disruption, driving pyroptosis.

*S*-Acylation predominantly functions by localizing proteins to specific membrane regions, thereby modulating their activity and interactions with other proteins and plays a critical role in apoptotic and non-apoptotic programmed cell death mechanisms, including necroptosis and pyroptosis, each defined by distinct molecular mechanisms and cellular outcomes. A common feature of these processes is the permeabilization of the cellular and plasma membrane. This permeabilization is accompanied by clearage by macrophages for apoptosis and inflammatory responses in the case of necroptosis and pyroptosis. *S*-Acylation has gained significant attention for its role in targeting key signaling proteins of these processes to membranes and contributing to the protein function and membrane integrity. In these pathways, especially during necroptosis and pyroptosis, lipidation can direct critical proteins involved in pore formation and membrane disruption to the plasma membrane, contributing to cell death mechanisms and the inflammatory activity associated with these processes (Figure 2).

### Multiple protein regulators of apoptosis are *S*-acylated

Apoptosis is one of the most widely studied forms of programmed cell death and is crucial for maintaining cellular homeostasis and development in multicellular organisms. This process involves a series of tightly regulated events that lead to cell shrinkage, chromatin condensation, membrane blebbing, and eventual fragmentation into apoptotic bodies, which are subsequently cleared by phagocytes. The initiation and execution of apoptosis are controlled by two main pathways: the intrinsic (mitochondrial) pathway and the extrinsic (death receptor) pathway. The intrinsic pathway is regulated by a balance between pro-apoptotic and anti-apoptotic members of the BCL-2 protein family, such as BAK (pro-apoptotic) and BCL-2 and BCL-XL (anti-apoptotic), which modulate mitochondrial outer membrane permeabilization. In contrast, the extrinsic pathway is triggered by the binding of death ligands to their respective cell surface receptors, such as FAS and TNF receptors, leading to the activation of caspase-8 and subsequent downstream caspase activation [14].

*S*-Acylation has emerged as an important post-translational modification affecting the localization, stability, and function of various apoptotic regulators, including both intrinsic and extrinsic apoptotic pathways [15]. This modification can influence the membrane association and signaling functions of apoptotic proteins. Notably, BAX has been found to undergo *S*-acylation, which modulates its activity and membrane targeting during apoptosis. *S*-Acylation of α5 helix at Cys 126 mediates the recruitment of BAX to mitochondrial membranes [16]. Once at the mitochondria, BAX causes the permeabilization of the mitochondrial outer membrane, releasing cytochrome c and initiating the activation of downstream caspases and the execution of the apoptotic cascade (Figure 2A). Importantly, mitochondrial BAX can be deacylated by APTs, highlighting the dynamic role of *S*-acylation in membrane recruitment and binding, modulating BAX-mediated mitochondrial membrane permeabilization [17].

The activation and function of caspases are primarily regulated through proteolytic processing and interactions with other proteins. While studies demonstrating *S*-acylation of caspases are limited, this lipid modification has the potential to influence their subcellular distribution and activation [18]. Specifically, *S*-acylation can impact the membrane association of certain caspases and their interactions with substrates, which in turn may affect their proteolytic activity. Notably, evidence linking *S*-acylation directly to caspase functioning during apoptosis remains scarce. However, a study has shown that caspase-6 can be *S*-acylated by the palmitoyltransferase zDHHC17. This *S*-acylation inhibits caspase-6 activation by preventing its substrate-binding and dimerization suggesting that *S*-acylation may serve as a regulatory mechanism to modulate caspase activity [19].

The FAS receptor (also known as CD95 or APO-1) is a key component of the extrinsic pathway of apoptosis. This receptor, a member of the tumor necrosis factor receptor superfamily, activates downstream signaling that leads to the formation of the death-inducing signaling complex and subsequent activation of initiator caspase-8. *S*-Acylation of the FAS receptor plays an important role in regulating its localization and function within the plasma membrane [20,21]. FAS *S*-palmitoylation occurs on Cys 199 close to the transmembrane domain and is catalyzed by zDHHC7 (Figure 2) [22]. Functionally, *S*-palmitoylation regulates FAS activity by mediating its association with certain membrane regions. This lipid modification facilitates the partitioning of FAS into specialized membrane microdomains enriched with cholesterol and sphingolipids, providing a platform for receptor clustering and death-inducing signaling complex assembly, enhancing apoptotic activity. By anchoring FAS receptor in these regions, *S*-acylation also helps maintain FAS levels at the plasma membrane preventing its degradation by the lysosomes [22]. Similar to BAX, reversible delipidation of FAS ensures that the receptor’s activity can be modulated in response to cellular conditions, potentially influencing the threshold for apoptosis induction.

### *S*-acylation of MLKL impacts its function in necroptosis

Necroptosis, or programmed necrosis, is a distinct form of non-apoptotic regulated cell death implicated in various diseases, including ischemia/reperfusion injuries, myocardial infarction, and cancer. It is also a driver for inflammatory responses associated with these conditions. Phenotypically, necroptosis is characterized by hallmarks of necrotic cell death, such as cell and organelle swelling and plasma membrane permeabilization. This permeabilization leads to the release of cytokines and other molecules into the extracellular matrix, triggering inflammation [23]. Recent studies have identified necroptosis as a target for developing new therapeutic strategies [23,24].

Necroptosis is primarily driven by the activity of receptor interacting protein kinases 1 and 3 (RIPK1/RIPK3). Under conditions of low caspase-8 activity, these kinases form a complex with MLKL. This complex phosphorylates MLKL, which oligomerizes and translocates to the plasma membrane, inducing permeabilization and cell death. MLKL’s interaction with the membrane is initially mediated by electrostatic interactions with membrane phospholipids [23].

Recent studies demonstrated that MLKL and its phosphorylated form (pMLKL) undergo *S*-acylation with palmitic acid and very long-chain fatty acids during necroptosis (Figure 2B) [3,4]. This modification enhances membrane binding of MLKL and exacerbates permeabilization via two key mechanisms. First, increased membrane binding facilitates membrane permeabilization [3]. Second, very long-chain fatty acylation induces local disruptions in lipid packing, promoting membrane destabilization. Investigations of *S*-palmitoyltransferases identified zDHHC21 as a critical enzyme for MLKL acylation. Inactivating zDHHC21 significantly reduced MLKL and pMLKL levels, improved membrane integrity, and destabilized protein at the membrane, leading to its degradation [3].

Interestingly, while MLKL and pMLK predominantly undergo single *S*-acylation, three cysteine residues (C184, C269, and C286 in human MLKL) are potential targets for this modification [3]. Molecular dynamics simulations suggest that these cysteines exhibit different propensities to interact with membrane lipids [3,25]. This mechanism proposes that acylation of one cysteine, coupled with the insertion of the acyl moiety into the membrane during necroptosis, may lock MLKL into a specific conformation. This locked state could, in turn, render the remaining cysteines less accessible for further acylation, thereby regulating the protein’s membrane-binding dynamics and downstream necroptotic activity. While these findings provide insight into the spatial and temporal regulation of MLKL by *S*-acylation, they are yet to be tested experimentally.

### Gasdermin D is *S*-acylated in pyroptosis

Pyroptosis is another highly inflammatory form of programmed cell death driven by gasdermin D, an effector protein of inflammasome signaling. Inflammasomes are innate immune complexes that respond to infections and cellular stress, leading to the activation of inflammatory caspases (caspase-1, -4, -5, and -11) and the release of proinflammatory cytokines IL-1β and IL-18. Upon activation, gasdermin D is cleaved by these caspases, which results in key conformational changes that separates its autoinhibitory C-terminal domain from its N-terminal domain. The released N-terminal domain undergoes additional conformational changes and translocates to the plasma membrane and oligomerizes. This process results in the release of intracellular contents, including cytokines and pro-inflammatory signal. While this process can play a protective role in fighting pathogens, its dysregulation is implicated in severe inflammatory conditions, including autoimmune diseases [26].

It is widely appreciated that activation of gasdermin D by caspases plays a pivotal role in transforming the N-terminal to an oligomeric pore-forming structure that translocates to the plasma membrane from an autoinhibited, soluble form [27]. Recent studies have revealed an additional mechanism that mediated pore-forming capacity of gasdermin D [28]. Balasubramanian et al. identified fatty acid synthase as a gasdermin D-binding partner and uncovered *S*-palmitoylation as a regulatory mechanism controlling gasdermin D activation. *S*-palmitoylation of gasdermin D at a conserved cysteine residue (Cys191 in humans), which is catalyzed by zDHHC5 and zDHHC9, mediates the membrane translocation of activated gasdermin D, but not full-length protein. Inhibition of gasdermin D palmitoylation suppresses pyroptosis and inflammatory response both *in vitro* and *in vivo*, mitigates organ damage, and improves survival in septic mice [29].

Further studies demonstrated that blocking gasdermin D acylation, either through mutagenesis or pharmacological inhibition, significantly reduced its palmitoylation, membrane localization, pyroptosis, and cytokine release. Conversely, co-expression of gasdermin D with a zDHHC enhanced pyroptotic activity and introducing exogenous palmitoylation sequences to gasdermin D Cys191 mutants restored functionality. These findings underscore the pivotal role of *S*-acylation in regulating gasdermin D activity during pyroptosis. Notably, *S*-acylation-mediated membrane recruitment of gasdermin D appears to be functionally distinct from other conformational changes associated with pyroptosis [30]. These results show *S*-palmitoylation as a key regulatory mechanism in pyroptotic activity and highlights its potential as a therapeutic target in infectious and inflammatory diseases.

While these studies have provided valuable mechanistic insights into how *S*-acylation contributes to cell death pathways, much remains to be uncovered regarding the regulation of cell death by *S*-acylation. For example, the activity of several zDHHCs has been linked to Toll-like receptor activation [31] and the modulation of apoptotic signaling (reviewed in [32]). A recent work of Ko et al*.* highlighted the critical role of *S*-acylation in regulating non-apoptotic cell death through an unknown mechanism [33]. The study identified zDHHC5 and Golgin subfamily A member 7 (GOLGA7), its accessory protein, as essential components of the non-apoptotic cell death pathway triggered by CIL56, a small molecule that induces non-canonical (non-apoptotic, non-necroptotic, or non-ferroptotic) cell death. They showed that the zDHHC5-GOLGA7 complex is stabilized through mutual interaction and localizes primarily at the plasma membrane, where the complex mediates *S*-acylation of downstream substrates critical for the cell death process. They also showed that loss of catalytic function or disruption of the zDHHC5-GOLGA7 complex significantly impairs the induction of cell death, highlighting that *S*-acylation of specific substrates is necessary for executing cell death. Mechanistically, *S*-acylation by ZDHHC5-GOLGA7 appears to regulate protein trafficking pathways critical for cell survival, particularly those involving retrograde transport from the Golgi apparatus to ER [33].

### Targeting *S*-acylation in disease contexts

Targeting *S*-acylation presents both opportunities and challenges in the modulation of cell death pathways, given its role in regulating proteins central to apoptosis, necroptosis, and pyroptosis. Below are some examples of the potential therapeutic consequences and outcomes of manipulating *S*-acylation for specific proteins involved in these pathways.

In apoptosis, inhibition of *S*-acylation of caspases can impact its proper localization to the death-inducing signaling complex. In cancers, this contributes to resistance to apoptotic stimuli and re-establishing proper *S*-acylation may overcome therapeutic resistance. Similarly, enhancing FAS S-acylation to stabilize its membrane clustering and death signal transduction can result in the activation of FAS and potentiate receptor-mediated cancer treatment. On the flip side, excessive activation of FAS through unregulated *S*-acylation might trigger off-target apoptotic events in healthy tissues, causing cytotoxicity [34].

In necroptosis and pyroptosis, *S*-acylation of MLKL and gasdermin D, respectively, modulate their role and can affect death signaling and membrane permeabilization associated with these processes. Enhancing MLKL *S*-acylation could restore necroptosis and cell death in apoptosis-resistant cancer cells. Conversely, reducing MLKL *S*-acylation may ameliorate membrane permeabilization [3] and tissue damage, and prevent pathological necroptosis observed in inflammatory diseases. Similarly, pharmacological intervention to modulate *S*-acylation of GSDMD can either enhance or prevent its membrane pore formation activity [29,30]. Inhibition of GSDMD *S*-acylation could prevent pyroptosis-mediated inflammatory damage in sepsis. Conversely, enhancing GSDMD activity via *S*-acylation might improve immune responses against certain intracellular bacterial infections. Overall, dysregulation of necroptosis [35] and pyroptosis [26] could either lead to excessive inflammation or impair immune clearance of infections, depending on the therapeutic direction.

Protein lipidation can also play an important role across various neurodegenerative diseases, including Huntington’s disease, through mechanisms not directly impacting cell death. One study showed that enhancing palmitoylation by pharmacologically inhibiting APT 1 effectively restored axonal transport, synapse homeostasis, and survival signaling. Increased S-acylation improved BDNF trafficking, reversed neuropathology, locomotor deficits, and anxio-depressive behaviors in disease models [36].

Targeting *S*-acylation and understanding its dynamic regulation present an exciting therapeutic frontier for modulating cell death pathways across diverse diseases. However, given the pleiotropic roles of *S*-acylation in cellular processes, precise targeting, ideally substrate-specific modulation, is essential to avoid off-target effects and unintended toxicity. Future research that focuses on understanding protein-specific *S*-acylation patterns and identifying pharmacological and genetic approaches that can regulate these modifications with high specificity will pave the way for new therapeutics on this front.

## Conclusions

*S*-Acylation influences a broad spectrum of proteins, extending its regulatory reach to scaffolding proteins, organelle homeostasis, and cytoskeletal dynamics, underscoring its versatility and widespread significance [37]. In this mini-review, we have explored the critical role of *S*-acylation in regulating key signaling proteins involved in apoptosis, necroptosis, and pyroptosis, highlighting its impact on membrane dynamics, protein localization, and functional activity during programmed cell death. Dysregulation of *S*-acylation disrupts protein function, subcellular localization, and signaling pathways, contributing to the pathogenesis of various diseases, including cancer, neurodegeneration, cardiovascular disorders, and infections. Therapeutically, targeting *S*-acylation is an emerging strategy, with pharmacological interventions aimed at modulating *S*-acyltransferase or acyl thioesterase activity showing promise in preclinical models. While small molecule inhibitors of *S*-acylation demonstrate potential to restore protein function and enhance cellular resilience, challenges remain in achieving selective targeting due to the conserved nature of active sites and the dynamic interplay within *S*-acylation networks.

Despite substantial progress, the full scope of *S*-acylation’s role in cell death and disease remains incompletely understood. The rapid advancement of proteomics and lipidomics continues to unveil new targets and lipid species involved in *S*-acylation, emphasizing the dynamic and evolving nature of this field. Along these lines, optimization of existing tool compounds and development of new generation compounds with enhanced potency and selectivity can further catalyze the developments on this front. Future research integrating structural biology, chemical biology, and systems-level analyses will be pivotal in unlocking the therapeutic potential of *S*-acylation modulation, providing a deeper understanding of its contributions to cell death and disease mechanisms.

**Perspective**

- Highlight importance of the field
  *S*-Acylation stands out among protein lipidation events for its ability to dynamically modulate protein localization and stability. It serves as a shared mechanism across multiple cell death pathways by targeting critical effectors at membranes. Notably, while historically linked to modification with palmitic acid, *S*-acylation also involves other fatty acids, broadening its impact on cellular processes and presenting new avenues for mechanistic exploration. By affecting protein distribution and membrane interactions, *S*-acylation exerts both spatial and temporal control over signaling events. Therapeutic strategies aimed at inhibiting *S*-acylation have demonstrated the potential to suppress cell death, underscoring *S*-acylation as a compelling target in cell death-related diseases.
- Summary of the current thinking
  Protein *S*-acylation plays a critical role in regulating protein localization, stability, and interactions with cellular membranes. While traditionally associated with palmitoylation, other fatty acids also contribute to *S*-acylation, expanding its regulatory potential. In apoptosis, *S*-acylation affects mitochondrial and death receptor signaling by modulating key proteins like Bcl-2-associated X and FAS. Similarly, *S*-acylation of mixed lineage kinase domain-like protein in necroptosis enhances membrane binding and pore formation, driving inflammatory responses. In pyroptosis, *S*-acylation of gasdermin D promotes pore assembly upon inflammasome activation, with palmitoylation inhibitors reducing inflammation and tissue damage. Overall, *S*-acylation emerges as a crucial regulator across cell death pathways, offering insights into the spatial and temporal control of protein function and membrane dynamics.
- Comment on future directions
  Although significant advancements have been made, the comprehensive role and scope of *S*-acylation in apoptosis, necroptosis, and pyroptosis is still not fully elucidated. Ongoing progress in proteomics and lipidomics will reveal new targets and lipid species linked to *S*-acylation. Integrating structural biology, chemical biology, systems-level approaches, and development of selective pharmacological interventions will be crucial for harnessing the biology, biochemistry, and physiology of *S*-acylation modulation and deepening our understanding of its role in cell death and disease pathways.

## Abbreviations

APTs: acyl-protein thioesterases
ER: endoplasmic reticulum
MLKL: mixed lineage kinase domain-like
